# Ophthalmitis, from Wearing a Dental Substitute Not Properly Adjusted

**Published:** 1853-01

**Authors:** H. L. Burpee

**Affiliations:** Suffield, Conn.


### ARTICLE VI.

### *Ophthalmitis, from Wearing a Dental Substitute not Properly Adjusted.* By H. L. Burpee, Suffield, Conn.

It is certainly of no small importance that a dental practitioner should fully and properly understand all the other organic functions of the human body, all its mechanism, all its connections of part with part, the individual action of every organ, its normal and morbid influence upon such other organs as are connected with it, and upon the whole economy; the sympathetic, as well as the organic laws which govern and control the whole physical system of man.

At the present stage of education in dental science it can be considered as little less than culpable negligence for an individual to undertake the practice of dental surgery without proper qualifications for its serious and arduous duties. It may be irrelevant to call to mind the festers and suppurating sores which afflict the profession at the present day, in consequence of malpractice by men who are well known to be quacks and empirics, but when we see a man, who bases his claims to public patronage on his boasted scientific medical education and fourteen or fifteen years experience in practical dentistry, fall into an error from which he cannot extricate himself, it should at least, serve as a landmark for the less experienced navigator.

From considerations like these, I have been induced to lay before the profession the following case, as presenting in itself some facts which are worthy of serious consideration.

Mrs. G. F., a highly respectable lady of this town, called on me in June last, for advice concerning a set of teeth, she was then, and had for a considerable time been, wearing, but still with great annoyance and inconvenience. From a few interrogations concerning the operations of the plate, as to her general health, &c. and without reluctance on her own part, the following facts were elicited, which have since been fully corroborated by her husband and attending physician. Mrs. F. has been somewhat enfeebled for a number of years by a chronic weakness of the spine, and a corresponding morbid condition of the nervous system, which had been greatly aggravated while wearing the teeth above mentioned.

In the spring of 1849, this dental substitute for the superior maxilla, had been constructed for her by a dentist from a neighboring town, some twelve miles distant,* (who comes this way frequently for the purpose of increasing his business,) in whom Mrs. F. reposed much confidence.

At the first trial the plate was not sufficiently well adjusted to prevent its being displaced by a slight touch, or by deglutition.

The patient's mouth, in consequence of the efforts of the dentist to make the teeth keep their place,† had become very sore and troublesome. Some alterations in the plate were made, and it was permitted to remain, drawing very hard by means of the vacuum formed in the plate. Notwithstanding the patient complained bitterly of the suffering caused by the plate drawing so hard, yet she was ordered to wear the teeth continually night and day, until she became accustomed to them, and the tenderness had subsided. A high state of inflammation was now manifestly extending over the entire surface of the gums and palatine arch,‡ and at this crisis of affairs, inflammation of the optic nerve commenced, and continued until chronic ophthalmia was established upon a tolerably firm basis.

Her physician evidently perceiving the real difficulty, saw there was danger of the patient becoming blind, and advised her not to wear the plate until refitted. This induced the patient to lay the substitute aside, although she did not wish to give her dentist unnecessary trouble.

When the dentist was informed of the sufferings of the patient, concerning the trouble about the eyes, &c. he discarded

* There was no resident dentist here at that time.

† By pushing and crowding the plate in the mouth to make it stick.

‡ The patient's mouth was actually blistered before the dentist left her.

the idea of the eyes and mouth having any connection whatever, saying that "*no one but a bloat would tell me so, let them prove it before they make such statement to me.*"

In the course of a little time, however, Mrs. F. and her husband succeeded in getting the work made over, and somewhat of the difficulty of the first plate was obviated, but not entirely, and from the time her eyes were first afflicted until I saw her, she had suffered almost constantly with the irritation caused by the plate sucking and moving about in the mouth. Whenever an attempt was made at mastication and a corresponding state of inflammation of the optic nerve, amounting at times to very great severity, so much so, that an entire loss of sight was at times greatly feared.

After a careful examination of the mouth and plate, I must say, I could see no reason why the plate had not been better fitted to say the least, and must confess myself not a little surprized that no impediment to a good fit presented itself, but it needed no herculian faculties of mind to discover that the plate in question could never be made to answer the purpose of its design. I advised a new plate of finer quality of gold, which I was employed to construct. The new substitute was soon adjusted and applied, and proves to be all that was required, and I think I may say much more than was anticipated.

The trouble about the eyes, has been gradually and decidedly improving ever since the new plate was inserted, and the soreness and tenderness of the gums and the palatine arch has entirely subsided, and the general health of the patient very greatly improved.

Here then it is plain, that in consequence of a badly fitted plate in the mouth, (or rather a *plate not fitted at all,*) a high state of inflammation of the gums and mucous membrane of the palatine arch was produced, and as the plate was continually worn, this state of affairs was constantly kept up. The superior maxillary nerve was necessarily involved, and by its connection with the ophthalmic branch at the ganglion of Casser, that nerve became similarly affected; and thus the eyes were made to suffer, whether by means of sympathetic laws or direct communication is a matter of too little moment to be discussed here. The diagnosis certainly appeared plain to me, that ophthalmia in this case was produced by the dental substitute before referred to.

I cannot leave this matter without a few passing remarks. It will doubtless excite surprise, that steps were not sooner taken to have the plate refitted, but if we examine the matter a little more fully, we shall manifest some degree of charity. When one person places implicit confidence in another, he is very much inclined to believe what that person tells him to be true. It often happens that confidence is misplaced and an individual grossly deceived, but the truth of the deception is admitted only from strong evidence and with caution. People are often deceived by empirics in the dental profession, but it is usually on a short acquaintance, and limited extent of the reputation of the operator, but in this case, the man had been known for years, and (in certain circles at least) enjoyed the reputation of being a good dentist and a responsible man, and by statements made, among others, was one very much like this, "that in consequence of certain peculiarities about the mouth, a good fit or even a comfortable fit could not be obtained." Mr. and Mrs. F. were made to believe, although against their better judgment, that their trouble arose from an inefficiency of the dental art, instead of their dentist.

But space will not allow me to enlarge, and I will close by one or two simple allusions. The "certain peculiarities" referred to by the venerable *doctor* (who by the way is loud in discarding quacks and young practitioners, and at the same time quite prolific of office students,) might in justice be noticed but for the fact, that I was not smart enough to find them in the mouth.

The *argument*, also, that the "eyes and mouth have no connection whatever" might deserve notice, could it be deemed of any importance to argue such a matter.

I submit the case, hoping that if any brother of the profession view the diagnosis in a different light, that I shall be corrected through the medium in which this article appears.

